# Lipase-Catalyzed Cyclization of β-Ketothioamides with β-Nitrostyrene for the Synthesis of Tetrasubstituted Dihydrothiophenes

**DOI:** 10.3390/molecules30153202

**Published:** 2025-07-30

**Authors:** Yihang Dai, Yuming Piao, Wenbo Kan, Lei Wang, Yazhuo Li

**Affiliations:** 1 Key Laboratory of Molecular Enzymology and Engineering of Ministry of Education, School of Life Sciences, Jilin University, Changchun 130023, China; 2 College of Food Science and Engineering, Jilin University, Changchun 130062, China

**Keywords:** tetrasubstituted dihydrothiophenes, porcine pancreatic lipase, environmentally friendly pathway, high yield, broad substrate scope, one-pot synthesis, simple post-processing

## Abstract

Tetrasubstituted dihydrothiophenes represent a class of heterocyclic compounds with significant potential in various fields, particularly in medicinal chemistry and materials science. In this work, we have developed an eco-friendly and efficient method for synthesizing such compounds, using porcine pancreatic lipase (PPL) as a biocatalyst to promote the cyclization reaction between β-ketothioamides and β-nitrostyrenes. Through this approach, sixteen tetrasubstituted dihydrothiophenes were successfully synthesized, and all of them achieved high yields, ranging from 80% to 96%. This research not only expands the application scope of lipase in organic synthesis, demonstrating its versatility beyond traditional hydrolytic reactions, but also provides a new environmentally friendly pathway for the production of tetrasubstituted dihydrothiophenes, which is of great significance for advancing related fields of chemical synthesis.

## 1. Introduction

**Scheme 1:**
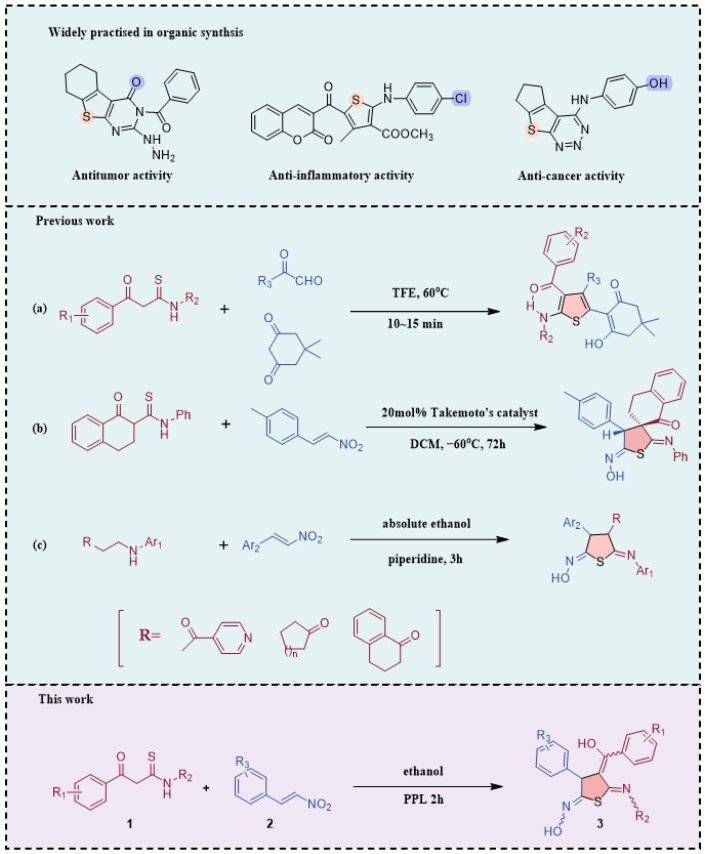
Previous works on the synthesis of tetrasubstituted thiophenes. (**a**) Synthesis of tetrasubstituted thiophenes using TFE as a catalyst; (**b**). Synthesis of chiral tetrasubstituted thiophenes using Takemoto’s catalyst in dichloromethane at −60 ^o^C; (**c**). Synthesis of tetrasubstituted thiophenes through various substitutions of KTA using piperidine as a catalyst.

Tetrasubstituted thiophenes, sulfur-containing five-membered heterocyclic compounds, are extensively utilized and have a crucial role in the fields of biomedicine and industrial production [1,2,3]. These compounds bear structural resemblances to specific natural compounds with therapeutic properties, showcasing distinctive biological activities and medicinal potential, such as being utilized as anti-inflammatory and anticancer agents (Scheme 1) [4,5,6,7]. As a result, they have become a focal point in the realm of drug synthesis investigation. The discovery and development of new thiophene-based drugs with pharmacological activities are of great significance for the progress of the medical and healthcare sector.

β-Ketothioamide (KTA) is a commonly employed building block in the synthesis of heterocyclic compounds [8,9,10]. Since the early 20th century, researchers have explored the potential of β-ketothioamide as a precursor for the production of tetrasubstituted thiophenes. In 1995, Bogdanowicz-Szwed’s research team successfully synthesized tetrasubstituted thiophenes by reacting KTA with β-nitrostyrene in ethanol with piperidine as a catalyst, resulting in high yields [11]. Subsequently, in 2001, 2003, 2004 and 2006, Bogdanowicz-Szwed’s team elaborated on the synthesis of tetrasubstituted thiophenes through various substitutions of KTA (Scheme 1c) [12,13,14,15]. In 2013, Wen’s group introduced a one-pot method for the synthesis of tetrasubstituted thiophenes using TFE as a catalyst, significantly reducing reaction time (Scheme 1a) [16]. Additionally, in 2015, Zeng et al. successfully synthesized chiral tetrasubstituted thiophenes at −60 °C using Takemoto’s catalyst in dichloromethane, achieving an enantioselectivity of 91% (Scheme 1b) [17]. Despite these advancements, the traditional synthesis of tetrasubstituted thiophenes often involves the use of hazardous reagents or environmentally harmful reaction conditions, such as piperidine and mercaptoacetaldehyde [18,19,20,21]. Therefore, there is a pressing need for the development of more sustainable and eco-friendly strategies in this field.

Lipases have been utilized in the catalysis of selective hydrolysis/transesterification of esters [22,23,24,25,26,27], kinetic resolution of α-substituted acids/alcohols with a chiral center. In addition, these enzymes have demonstrated the ability to catalyze various organic reactions such as aldol reaction, Michael reaction, and Knoevenagel reaction [28,29,30,31,32] to yield corresponding heterocyclic compounds under mild conditions. Despite this, lipases are seldom employed in the construction of thiophene motifs, which serve as widely used, cost-effective, and versatile building blocks. Given the potential enzymatic applications in green chemistry and sustainable pharmaceutical syntheses [33,34,35,36,37,38,39,40], further research on lipases is warranted and holds significant interest. In this work, an enzymatic method for synthesizing tetrasubstituted dihydrothiophenes via the cyclization of β-ketothioamide and β-nitrostyrene has been reported for the first time.

## 2. Results and Discussion

**Scheme 2:**
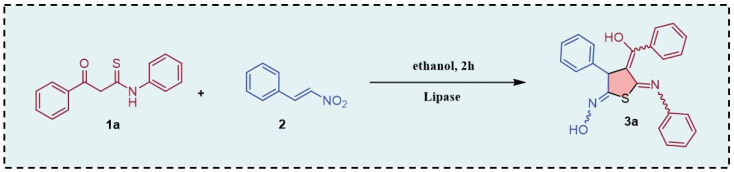
The model reaction catalyzed by lipase.

To establish an efficient and sustainable protocol for the synthesis of tetrasubstituted dihydrothiophenes, we first sought to identify the optimal biocatalyst by evaluating the catalytic performance of lipases from diverse sources, using the cyclization of β-ketothioamide (KTA **1a**) and β-nitrostyrene **2** to form tetrasubstituted dihydrothiophene **3a** as a model reaction (Scheme 2). A systematic screening of lipases from various origins revealed that all tested lipases exhibited detectable catalytic activity toward the model substrates (entries 1–6, Table 1). Among these, porcine pancreatic lipase (PPL) stood out with the highest catalytic efficiency, affording **3a** in a remarkable yield of 92%. The varying catalytic activities observed for other lipases (e.g., *Candida* sp. lipase (CSL) with 46% yield, *C. rugosa* lipase (CRL) with 55% yield) are likely attributed to distinct structural features in their active sites or overall protein conformations, which govern substrate binding affinity and catalytic turnover rates. To rigorously validate that the catalytic activity stems from the enzymatic function of lipases rather than non-specific protein effects, control experiments were performed. Substituting lipase with bovine serum albumin (BSA)—a non-enzymatic protein—resulted in no detectable formation of **3a** (entry 7), ruling out the possibility of non-catalytic protein-mediated reactions. Further, heat-denatured PPL (treated at 100 °C for 12 h) failed to yield the target product (entry 8), indicating that the native protein conformation is indispensable for catalytic activity. To pinpoint the role of the active center, PPL was inactivated using phenylmethanesulfonyl fluoride (PMSF), a specific inhibitor of serine hydrolases that covalently modifies the catalytic serine residue in the active site. As expected, this PMSF-inactivated PPL yielded no **3a** (entry 9), mirroring the outcome of the blank control (no catalyst, entry 10). Collectively, these results confirm that the catalytic activity is dependent on both the intact active center of the lipase and its native conformational state, with PPL’s unique structural features enabling its superior performance. For comparison, we also evaluated triethylamine—a commonly used chemical catalyst—in the model reaction. The yield of **3a** was significantly lower (53%, entry 11) than that achieved with PPL, underscoring the superiority of the enzymatic approach. Notably, the stoichiometric amount of triethylamine required (equimolar to the substrate) contrasts with the catalytic amount of PPL (40 U), further highlighting the superior efficiency and atom economy of the enzymatic method. These findings reinforce the potential of PPL as a robust biocatalyst for the synthesis of tetrasubstituted dihydrothiophenes, aligning with the principles of green chemistry.

Solvent polarity is a crucial factor to consider in organic synthesis reactions, and in this research, we evaluated the performance of eight solvents with varying polarities, as outlined in Table 2, to identify the most suitable reaction medium for the lipase-catalyzed cyclization system. The findings revealed a distinct trend: polar solvents such as ethanol and acetonitrile displayed superior reaction efficiency (entries 2–3), while non-polar solvents including dichloromethane and n-hexane yielded less favorable results (entries 7–8), indicating a clear correlation between solvent polarity and reaction performance. Despite water being recognized as an environmentally friendly polar solvent, its minor substrate solubility and the resulting low reaction yield led us to exclude it from consideration. Although acetonitrile provided higher product yields, we chose ethanol as the reaction solvent because it is more in keeping with green chemistry principles, and after thorough deliberation, ethanol was ultimately selected as the optimal reaction solvent.

**Figure 1:**
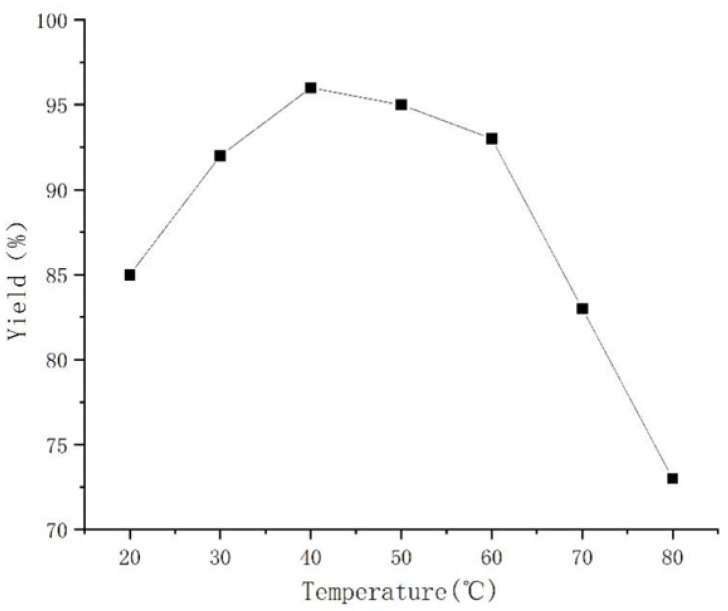
Effect of temperature on the enzymatic synthesis of tetrasubstituted dihydrothiophene (**3a**). Reaction conditions: **1a** (0.2 mmol), **2** (0.2 mmol), EtOH (1 mL), PPL (40 U, powder), 2 h.

In enzymatic reactions, temperature plays an essential role in determining the catalytic efficiency of enzymes. To explore the optimal temperature, we conducted experiments in the range of 20 °C to 80 °C (Figure 1). The maximum yield was at 40 °C. From 50 °C to 60 °C, the yield decreases but is still superior to 30 °C. At higher temperatures, the yield significantly decreases. Such a result is related to two key factors: molecular thermal movement and protein structure. Elevated temperatures enhance molecular thermal movement, while excessively high temperatures can cause protein denaturation, thereby impeding catalytic efficiency. Notably, we achieved a reaction yield of 92% at 30 °C. Although a higher yield of 96% was achieved at 40 °C, we ultimately selected 30 °C as the optimal reaction temperature, taking into account energy consumption and experimental convenience.

**Figure 2:**
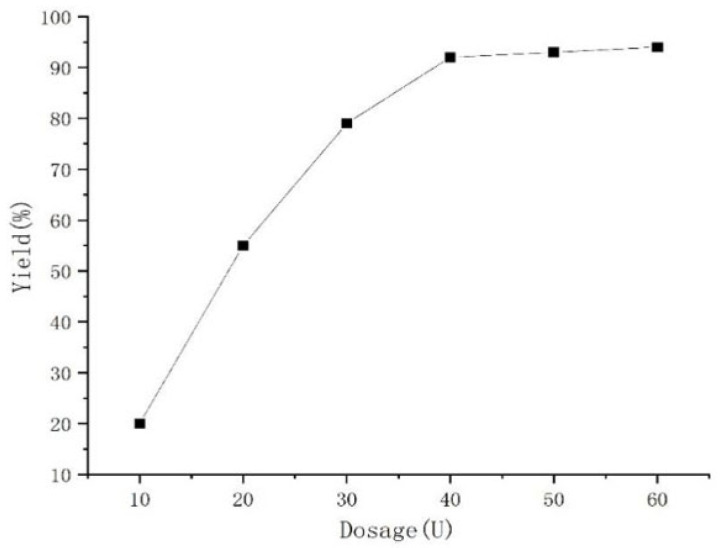
Effect of enzyme dosage on the enzymatic synthesis of tetrasubstituted dihydrothiophene (**3a**). Reaction conditions: **1a** (0.2 mmol), **2** (0.2 mmol), EtOH (1 mL), 30 °C, 2 h.

Subsequent experiments were performed to determine the optimal enzyme dosage for the reaction, and as depicted in Figure 2, a significant improvement in product yield was observed with increasing enzyme concentration, with the yield reaching its peak at an enzyme dosage of 40 U. Further increments in enzyme dosage beyond this threshold failed to result in a corresponding increase in yield. This phenomenon can be plausibly attributed to the system attaining a state of thermodynamic equilibrium after the two-hour reaction period, where the concentration of active enzyme no longer acts as a limiting factor for the reaction progress. This observation not only identifies 40 U as the optimal enzyme dosage for maximizing the synthesis efficiency of tetrasubstituted dihydrothiophenes under the given reaction conditions but also underscores the importance of balancing catalyst loading with reaction kinetics to avoid unnecessary resource consumption, aligning with the principles of green chemistry emphasized in this study.

**Scheme 3:**
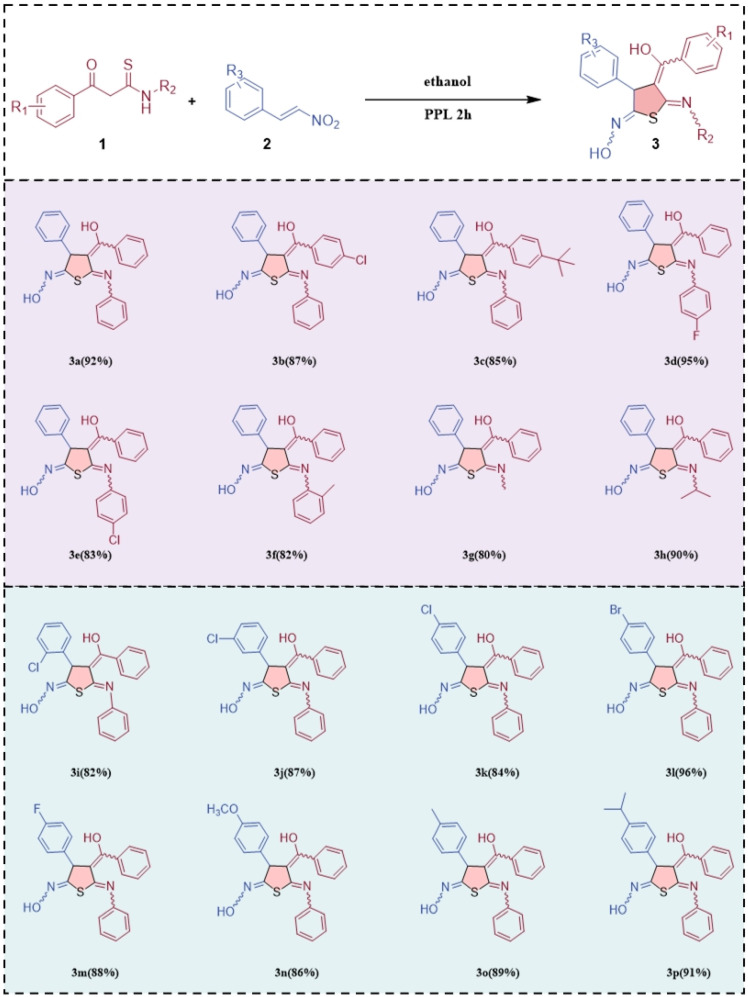
Synthesis of tetrasubstituted thiophenes **3** catalyzed by lipase. Reaction conditions: **1** (0.2 mmol), **2** (0.2 mmol), EtOH (1 mL), PPL (40 U, powder), 30 °C, 2 h.

After confirming the optimal reaction conditions, we proceeded with investigating substrate diversity. As illustrated in Scheme 3, the yield of para-substituted substrates (**3a**–**3c**) on the benzene ring (R_1_) of compound **1** ranged from 85% to 92%. Moreover, the nitrogen atom (R_2_) of **1**, whether linked to an aryl group with significant steric hindrance or a methyl group with minimal steric hindrance (**3c**–**3h**), consistently achieved high yields, ranging from 80% to 95%. Regardless of *para*-, *meta*-, or *ortho*-substitutions (**3i**–**3k**), electron-donating groups, or electron-withdrawing groups (**3k**–**3p**) on the benzene ring (R_3_) of compound **2**, the outcomes remained significant, ranging from 82% to 96%. These findings highlight the broad substrate applicability in the synthesis of tetrasubstituted dihydrothiophenes catalyzed by PPL.

**Scheme 4:**
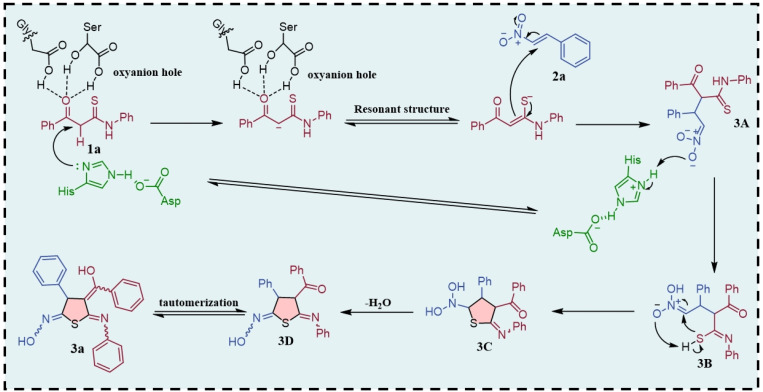
Proposed mechanism for lipase synthesis of tetrasubstituted dihydrothiophene.

Building upon prior investigations into the reaction mechanisms underlying lipase-catalyzed analogous transformations [41,42,43] and the structure of PPL [44], we propose a potential mechanism for the enzymatic reaction as follows (Scheme 4): **1a** is stabilized by the oxyanion hole (including Ser in the catalytic triad of lipase), then undergoes deprotonation by lipase, leading to the formation of an anion that subsequently participates in an addition with substrate **2a** (this structure is stabilized by resonance with the nitro group), resulting in the formation of intermediate **3A**. The intermediate **3A** is then converted into **3B** through protonation, and the histidine residue of the enzyme reverts to its original conformation. Then, **3B** is converted into the 5-membered heterocyclic compound **3C** through a nucleophilic attack involving the sulfur atom on the carbon atom. The dehydration of **3C** leads to the production of tetrasubstituted dihydrothiophene **3D**. Subsequently, the tautomeric intermediate **3D** undergoes tautomerization to form **3a**. (More details of the products, please see in Supplementary Materials).

## 3. Materials and Methods

### 3.1. General Information

PPL (Porcine pancreatic lipase, 5600 U/g), CSL (Lipase from *Candida* sp., 6400 U/g) was purchased from Shanghai Yuan Ye Biological Technology Company (Shanghai, China), CRL (C. *rugose* lipase), MML (*Mucor mieei* lipase, 7300 U/g), Cal-B (*C. antarctica* lipase B, 10,000 U/mL), BSA (bovine serum albumin) were purchased from Sigma-Aldrich China Co. (Beijing, China). Novozym 435 (15,000 U/g) was purchased from Novo Nordisk Co., Ltd. (Beijing, China). One unit of enzyme activity is defined as the quantity of enzyme necessary to catalyze the hydrolysis of 1 μmol p-nitrophenyl acetate per minute at 30 °C. β-Ketothioamides were prepared in the laboratory. β-Nitrostyrene, ethanol and all other chemical reagents were bought from Bide Pharmatech. Ltd. and Energy-Chemical Ltd. (Shanghai, China). Silica gel chromatography purifications were carried out using AMD Silica Gel 60 230–400 mesh. Thin Layer Chromatography (TLC) and preparative TLC were carried out using TLC silica gel 60 F254 glass plates (BM000035), which were purchased from Energy-Chemical Ltd. (Shanghai, China). All commercially available reagents and solvents were used as received without additional purification. The NMR spectrometer was purchased from Bruker (AVANCEIII 500) (Thermo Scientific, San Jose, CA, USA). Proton nuclear magnetic resonance (^1^H NMR) spectra were recorded on a 400 MHz spectrometer in DMSO-d6, carbon nuclear magnetic resonance (^13^C NMR) spectra were recorded on a 101 MHz spectrometer in DMSO-d6. Chemical shifts for protons are reported in parts per million downfield from tetramethylsilane (TMS) and are referenced to residual protium in the NMR solvent (DMSO-d6 = δ 2.50 ppm). Chemical shifts for carbon are reported in parts per million downfield from tetramethylsilane (TMS) and are referenced to the carbon resonances of the solvent residual peak (DMSO-d6 = δ 39.6 ppm). NMR data are presented as follows: chemical shift (δ ppm), multiplicity (s = singlet, d = doublet, t = triplet, q = quartet, m = multiplet), coupling constant in Hertz (Hz), integration. Mass spectra were recorded on the Bruker MicrOTOF Q II and an Orbitrap FusionTM TribridTM mass spectrometer (Thermo Scientific, San Jose, CA, USA) coupled with HESI ion source.

### 3.2. General Procedure for Synthesis of β-Ketothioamide (**1**)

40 mmol of the corresponding acetophenone was dissolved in 30 mL 1,4-dioxane, and 40 mmol NaH was added. At 30 °C, the corresponding aryl or alkyl isothiocyanate (40 mmol) was slowly added while stirring, and stirring was continued at 30 °C for 2 h. After cooling to room temperature, the solids were filtered, washed with 1,4-dioxane and dissolved in water. The solution was slowly neutralized with dilute HCl, the precipitate was isolated by filtration and dried. The obtained substances 1 do not require further purification and can be directly used in the subsequent steps [45].

### 3.3. General Procedure for Lipase-Catalyzed Synthesis of Tetrasubstituted Dihydrothiophenes (**3**)

β-ketothioamide **1** (0.2 mmol) and β-nitrostyrene **2** (0.2 mmol) were dissolved in ethanol (1 mL), and PPL (powder, 40 U) was added. The mixture was stirred at 30 °C for 2 h. After verifying the reaction with TLC (silica with petroleum ether/ethyl acetate = 3:1, *v*/*v*), the solvent was evaporated, and the residue was submitted to column chromatography (silica with petroleum ether/ethyl acetate = 3:1, *v*/*v*), resulting in pure solid products 3.

## 4. Conclusions

In this study, we have successfully established a highly efficient enzymatic approach for the synthesis of tetrasubstituted dihydrothiophenes, which proceeds via lipase-catalyzed cyclization of β-ketothioamides with β-nitrostyrenes. This method not only boasts notable advantages such as straightforward post-treatment and environmental benignity but also achieves impressive yields spanning 80% to 96% across a diverse range of substrate derivatives, thereby underscoring its robust efficiency and broad substrate scope. By utilizing porcine pancreatic lipase (PPL) as the biocatalyst and ethanol as the reaction medium, this strategy eliminates the need for environmentally hazardous reagents—including piperidine, dichloromethane, and transition metal catalysts—that are frequently employed in conventional chemical synthesis protocols; these reagents, with their inherent toxicity and persistence, present substantial obstacles to ecological sustainability. Aligned with the fundamental principles of green chemistry, this enzymatic approach not only extends the catalytic repertoire of lipases beyond their typical hydrolytic and transesterification reactions but also establishes a novel, sustainable synthetic framework for accessing tetrasubstituted dihydrothiophenes.

## Figures and Tables

**Table 1:** The effect of enzyme origin on the synthesis of tetrasubstituted dihydrothiophene (**3a**).

Entry	Catalyst	Yield (%)
1	PPL ^1^	92
2	CSL	46
3	CRL	55
4	MML	52
5	Cal-B	68
6	Movozym-435	76
7	BSA	N.D.
8	PPL ^2^	N.D.
9	PPL ^3^	N.D.
10	None	N.D.
11	Triethylamine ^4^	53

Reaction conditions: **1a** (0.2 mmol), **2** (0.2 mmol), ethanol (1 mL), lipase (40 U), 30 °C, 2 h. PPL ^1^ (Porcine pancreatic lipase); CSL (*Candida* sp. lipase); CRL (*C. rugosa* lipase); MML (*Mucor miehei* Lipase); Cal-B (*C. antarctica* lipase B); Novozym 435 (a commercial immobilized Cal-B); BSA (bovine serum albumin); PPL ^2^ was denatured by heating it to 100 °C for 12 h in water; PPL ^3^ was inactivated by phenylmethanesulfonyl fluoride (PMSF, an irreversible inhibitor); ^4^ Triethylamine (0.2 mmol); N.D.: not found.

**Table 2:** Effect of solvents on the enzymatic synthesis of tetrasubstituted dihydrothiophene (**3a**).

Entry	Solvent	Yield (%)
1	Water	80
2	Ethanol	92
3	Acetonitrile	94
4	Dimethyl sulfoxide	67
5	*N*, *N*-dimethylformamide	76
6	Ethyl acetate	84
7	Dichloromethane	50
8	n-Hexane	43

Reaction conditions: **1a** (0.2 mmol), **2** (0.2 mmol), solvent (1 mL), PPL (40 U, powder), 30 °C, 2 h.

## Data Availability

Data are contained within the article and Supplementary Materials.

## Supplementary Materials

The following supporting information can be downloaded at: https://www.mdpi.com/article/10.3390/molecules30153202/s1, S1: ^1^H NMR and MS data of products **3**, ^13^C NMR data of products **3a** and **3b**; S2: ^1^H NMR spectra of products **3** and ^13^C NMR spectrum of products **3a** and **3b**; S3: mass spectrum of product **3a**.
