# Supplementary material for: Lipase-Catalyzed Cyclization of β-Ketothioamides with β-Nitrostyrene for the Synthesis of Tetrasubstituted Dihydrothiophenes
Source: Molecules. 2025 Jul 30;30(15):3202. doi: 10.3390/molecules30153202 (PMC12348498; doi:10.3390/molecules30153202)

Supporting Information for

Lipase-catalyzed cyclization of β-ketothioamides with β-nitrostyrene for the synthesis of tetrasubstituted dihydrothiophenes

Yihang Dai [1], Yuming Piao [1], Wenbo Kan [1], Lei Wang [1, *], Yazhuo Li [2, *]

[1] Key Laboratory of Molecular Enzymology and Engineering of Ministry of Education, School of Life Sciences, Jilin University, Changchun 130023, China; daiyh1323@mails.jlu.edu.cn (Y.D.); piaoym1321@mails.jlu.edu.cn (Y.P.); kanwb1321@mails.jlu.edu.cn (W.K.)

[2] College of Food Science and Engineering, Jilin University, Changchun 130062, China

* Correspondence: w_lei@jlu.edu.cn (L.W.); LLL@jlu.edu.cn (Y.L.)

Table of Contents

S1. Data of Products 2

S2. ^{1}H NMR and ^{13}C NMR of Products 6

S3. Mass spectrum of 3a 15

S1. Data of Products

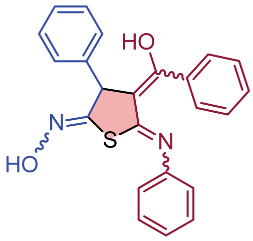

3a 4-(hydroxy(phenyl)methylene)-3-phenyl-5-(phenylimino)dihydrothiophen-2(3H)-one oxime.

Isolated yield: 92% (71.04 mg), yellow solid, ^{1}H NMR (400 MHz, DMSO-d6) δ 12.73 (s, 1H), 11.66 (s, 1H), 7.47 (s, 2H), 7.24 – 7.29 (m, 5H), 7.23 (t, J = 7.5 Hz, 4H), 7.06 (s, 2H), 6.88 (d, J = 7.6 Hz, 2H), 5.51(s, 1H). ^{13}C NMR (101MHz, DMSO-d6) δ 190.47, 162.92, 157.72, 141.80, 141.56, 139.52, 129.66, 129.34, 128.39, 128.00, 127.23, 126.83, 126.37, 125.88, 123.08, 106.10, 55.33. MS (ESI): m/z = $[M + H]^+$ calcd for $(C_{23}H_{18}N_2O_2S)H^+$, 387.1089; found, 387.1091.

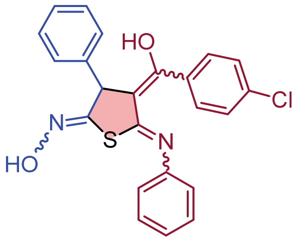

3b 4-((4-chlorophenyl)(hydroxy)methylene)-3-phenyl-5-(phenylimino)dihydrothiophen-2(3H)-one oxime.

Isolated yield: 87% (73.10 mg), yellow solid, ^{1}H NMR (400 MHz, DMSO-d6) δ 12.69 (s, 1H), 11.68 (s, 1H), 7.48 (s, 2H), 7.32 (s, 2H), 7.28 (d, J = 5.2 Hz, 2 H), 7.09 – 7.13 (m, 4H), 6.93 (t, J = 8.6 Hz, 4H), 5.53 (s, 1H). ^{13}C NMR (101MHz, DMSO-d6) δ 191.91, 162.95, 158.69, 145.14, 141.37, 138.51, 130.11, 129.66, 128.85, 128.39, 128.00, 127.65, 126.85, 126.43, 123.47, 114.81, 53.46. MS (ESI): m/z = $[M + H]^+$ calcd for $(C_{23}H_{17}ClN_2O_2S)H^+$, 421.0699; found, 421.0697.

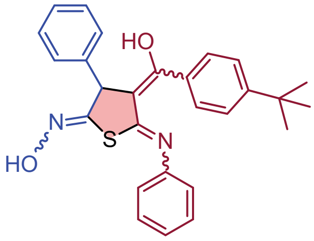

3c 4-((4-(tert-butyl)phenyl)(hydroxy)methylene)-3-phenyl-5-(phenylimino)dihydrothiophen-2(3H)-one oxime.

Isolated yield: 85% (75.17 mg), yellow solid, ^{1}H NMR (400 MHz, DMSO-d6) δ 12.75 (s, 1H), 11.66 (s, 1H), 7.49 – 7.43 (m, 4H), 7.31 (dd, J = 7.8, 4.3 Hz, 2H), 7.26 – 7.21 (m, 4H), 7.06 – 7.03 (m, 2H), 6.85 (d, J = 3.8 Hz, 2H), 5.48 (s, 1H), 1.24 (s, 9H). MS (ESI): m/z = $[M + H]^+$ calcd for $(C_{27}H_{26}N_2O_2S)H^+$, 444.1715; found, 444.1716.

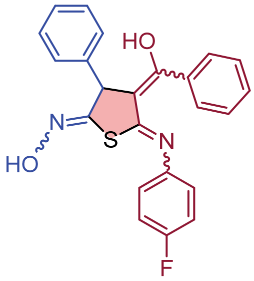

3d 5-((4-fluorophenyl)imino)-4-(hydroxy(phenyl)methylene)-3-phenyl dihydrothiophen-2(3H)-one oxime.

Isolated yield: 95% (76.78 mg), yellow solid, ^{1}H NMR (400 MHz, DMSO-d6) δ 12.47 (s, 1H), 11.39 (s, 1H), 7.53 (dd, J = 8.6, 4.9 Hz, 3H), 7.29 – 7.35 (m, 4H), 7.21 (d, J = 7.5 Hz, 2H), 7.06 (s, 4H), 6.90 (d, J = 5.8 Hz, 1H), 5.56 (s, 1H). MS (ESI): m/z = $[M + H]^+$ calcd for $(C_{23}H_{17}FN_2O_2S)H^+$, 405.0995; found, 405.0994.

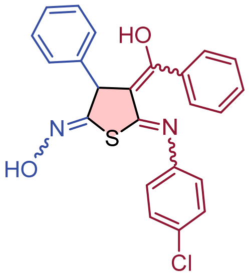

3e 5-((4-chlorophenyl)imino)-4-(hydroxy(phenyl)methylene)-3-phenyl dihydrothiophen-2(3H)-one oxime.

Isolated yield: 83% (69.74 mg), yellow solid, ^{1}H NMR (400 MHz, DMSO-d6) δ 12.56 (s, 1H), 11.42 (s, 1H), 7.52 (s, 2H), 7.31 – 7.21 (m, 5H), 7.07 (d, J = 6.4 Hz, 4H), 6.89 (t, J = 7.6 Hz, 3H), 5.56 (s, 1H). MS (ESI): m/z = $[M + H]^+$ calcd for $(C_{23}H_{17}ClN_2O_2S)H^+$, 421.0699; found, 421.0697.

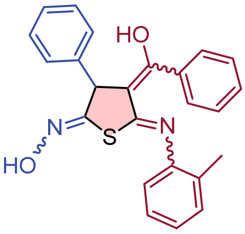

3f 4-(hydroxy(phenyl)methylene)-3-phenyl-5-(o-tolylimino)dihydrothiophen-2(3H)-one oxime.

Isolated yield: 82% (65.62 mg), yellow solid, ^{1}H NMR (400 MHz, DMSO-d6) δ 12.65 (s, 1H), 11.62 (s, 1H), 7.47 (d, J = 7.6 Hz, 2H), 7.30 (s, 2H), 7.25 (s, 2H), 7.08 (d, J = 8.0 Hz, 5H), 6.88 (d, J = 7.7 Hz, 2H), 5.53 (s, 1H), 2.40(s, 3H). MS (ESI): m/z = $[M + H]^+$ calcd for $(C_{24}H_{20}N_2O_2S)H^+$, 401.1245; found, 401.1246.

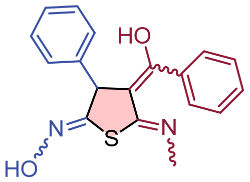

3g 4-(hydroxy(phenyl)methylene)-5-(methylimino)-3-phenyldihydrothiophen-2(3H)-one oxime.

Isolated yield: 80% (51.86 mg), yellow solid, ^{1}H NMR (400 MHz, DMSO-d6) δ 11.67 – 11.62 (m, 1H), 10.67 (s, 1H), 7.27 – 7.13 (m, 5H), 7.04 (d, J = 7.5 Hz, 3H), 6.81 (d, J = 7.0 Hz, 2H), 5.38 (s, 1H), 3.14 (d, J = 4.9 Hz, 3H). MS (ESI): m/z = $[M + H]^+$ calcd for $(C_{18}H_{16}N_2O_2S)H^+$, 325.0932; found, 325.0931.

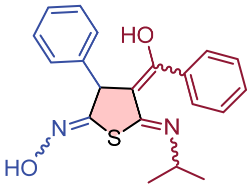

3h 4-(hydroxy(phenyl)methylene)-5-(isopropylimino)-3-phenyldihydrothiophen-2(3H)-one oxime.

Isolated yield: 90% (63.38 mg), yellow solid, ^{1}H NMR (400 MHz, DMSO-d6) δ 11.66 (s, 1H), 11.00 (d, J = 8.6 Hz, 1H), 7.26 – 7.21 (m, 2H), 7.19 – 7.16 (m, 4H), 7.04 (d, J = 7.8 Hz, 2H), 6.79 (d, J = 6.9 Hz, 2H), 5.38 (s, 1H), 3.78 – 3.61 (m, 1H), 1.37 (d, J = 6.3 Hz, 3H), 1.32 (d, J = 5.3 Hz, 3H). MS (ESI): m/z = $[M + H]^+$ calcd for $(C_{20}H_{20}N_2O_2S)H^+$, 353.1245; found, 353.1246.

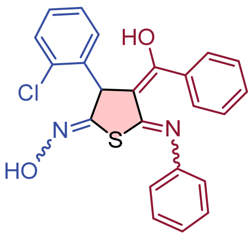

3i 3-(2-chlorophenyl)-4-(hydroxy(phenyl)methylene)-5-(phenylimino) dihydrothiophen-2(3H)-one oxime.

Isolated yield: 82% (68.90 mg), yellow solid, ^{1}H NMR (400 MHz, DMSO-d6) δ 12.73 (s, 1H), 11.74 (s, 1H), 7.51 – 7.43 (m, 5H), 7.32 (t, J = 6.5 Hz, 1H), 7.27 – 7.17 (m, 5H), 7.14 – 7.07 (m, 3H), 6.01 (s, 1H). MS (ESI): m/z = $[M + H]^+$ calcd for $(C_{23}H_{17}ClN_2O_2S)H^+$, 421.0699; found, 421.0697.

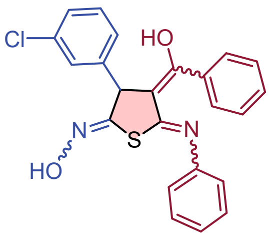

3j 3-(3-chlorophenyl)-4-(hydroxy(phenyl)methylene)-5-(phenylimino)dihydrothiophen-2(3H)-one oxime.

Isolated yield: 87% (73.10 mg), yellow solid, ^{1}H NMR (400 MHz, DMSO-d6) δ 12.62 (s, 1H), 11.76 (s, 1H), 7.48 (s, 3H), 7.31 (s, 3H), 7.27 – 7.21 (m, 4H), 7.11 (d, J = 4.5 Hz, 4H), 5.58 (s, 1H). MS (ESI): m/z = [M + H]$^+$ calcd for ($C_{23}H_{17}ClN_2O_2S$)H$^+$, 421.0699; found, 421.0698.

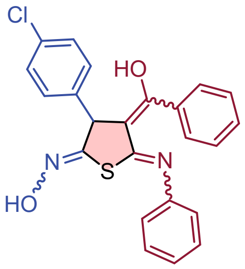

3k 3-(4-chlorophenyl)-4-(hydroxy(phenyl)methylene)-5-(phenylimino)dihydrothiophen-2(3H)-one oxime.

Isolated yield: 84% (70.58 mg), yellow solid, ^{1}H NMR (400 MHz, DMSO-d6) δ 12.71 (s, 1H), 11.47 (s, 1H), 7.49 (s, 2H), 7.31 (d, J = 5.0 Hz, 3H), 7.25 (dd, J = 7.8, 4.0 Hz, 4H), 7.15 – 7.08 (m, 3H), 6.89 (d, J = 3.3 Hz, 2H), 5.76 (s, 1H). MS (ESI): m/z = [M + H]$^+$ calcd for ($C_{23}H_{17}ClN_2O_2S$)H$^+$, 421.0699; found, 421.0695.

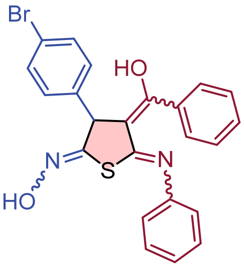

3l 3-(4-bromophenyl)-4-(hydroxy(phenyl)methylene)-5-(phenylimino)dihydrothiophen-2(3H)-one oxime.

Isolated yield: 96% (89.09 mg), yellow solid, ^{1}H NMR (400 MHz, DMSO-d6) δ 12.72 (s, 1H), 11.74 (s, 1H), 7.45 – 7.49 (m, 4H), 7.31 (s, 3H), 7.23 – 7.26 (m, 4H), 6.83 (d, J = 8.5 Hz, 3H), 5.56 (s, 1H). MS (ESI): m/z = [M + H]$^+$ calcd for ($C_{23}H_{17}BrN_2O_2S$)H$^+$ 465.0194; found, 465.0195.

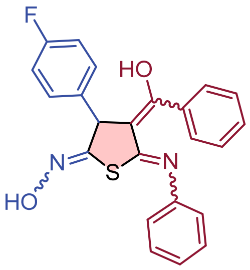

3m 3-(4-fluorophenyl)-4-(hydroxy(phenyl)methylene)-5-(phenylimino)dihydrothiophen-2(3H)-one oxime.

Isolated yield: 88% (71.12 mg), yellow solid, ^{1}H NMR (400 MHz, DMSO-d6) δ 12.67 (s, 1H), 11.44 (s, 1H), 7.47 (s, 2H), 7.29 – 7.33 (m, 5H), 7.23 (d, J = 7.3 Hz, 2H), 6.89 (d, J = 7.1 Hz, 5H), 5.76 (s, 1H). MS (ESI): m/z = [M + H]$^+$ calcd for ($C_{23}H_{17}FN_2O_2S$)H$^+$, 405.0995; found, 405.0996.

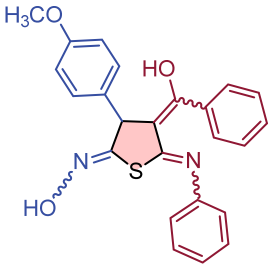

3n 4-(hydroxy(phenyl)methylene)-3-(4-methoxyphenyl)-5-(phenylimino)dihydrothiophen-2(3H)-one oxime.

Isolated yield: 86% (71.57 mg), yellow solid, ^{1}H NMR (400 MHz, DMSO-d6) δ 12.71 (s, 1H), 11.39 (s, 1H), 7.47 (s, 2H), 7.32 (d, J = 6.8 Hz, 5H), 7.24 (d, J = 7.6 Hz, 3H), 6.81 (dd, J = 12.4, 8.2 Hz, 2H), 6.64 (d, J = 8.2 Hz, 2H), 5.76 (s, 1H), 3.64 (s, 3H). MS (ESI): m/z = $[M + H]^+$ calcd for $(C_{24}H_{20}N_2O_3S)H^+$, 417.1195; found, 417.1197.

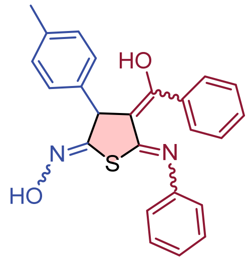

3o 4-(hydroxy(phenyl)methylene)-5-(phenylimino)-3-(p-tolyl)dihydrothiophen-2(3H)-one oxime.

Isolated yield: 89% (71.22 mg), yellow solid, ^{1}H NMR (400 MHz, DMSO-d6) δ 12.73 (s, 1H), 11.64 (s, 1H), 7.46 (d, J = 3.5 Hz, 2H), 7.31 (s, 3H), 7.25 (d, J = 6.1 Hz, 2H), 6.88 (s, 3H), 6.78 (t, J = 8.4 Hz, 4H), 5.76 (s, 1H), 2.15 (d, J = 6.0 Hz, 3H). MS (ESI): m/z = $[M + H]^+$ calcd for $(C_{24}H_{20}N_2O_2S)H^+$, 401.1245; found, 401.1247.

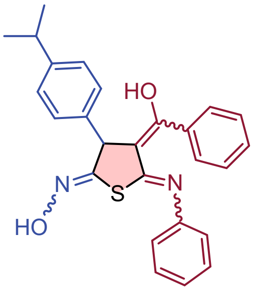

3p 4-(hydroxy(phenyl)methylene)-3-(4-isopropylphenyl)-5-(phenylimino)dihydrothiophen-2(3H)-one oxime.

Isolated yield: 91% (77.93 mg), yellow solid, ^{1}H NMR (400 MHz, DMSO-d6) δ 12.70 (s, 1H), 11.65 (s, 1H), 7.45 (s, 2H), 7.29 – 7.34 (m, 5H), 7.22 (s, 2H), 6.94 (d, J = 7.7 Hz, 3H), 6.80 (d, 2H), 5.44 (s, 1H), 2.73 (p, J = 6.9 Hz, 1H), 1.09 (q, 6H). MS (ESI): m/z = $[M + H]^+$ calcd for $(C_{26}H_{24}N_2O_2S)H^+$, 429.1558; found, 429.1559.

S2. ^{1}H NMR and ^{13}C NMR of Products

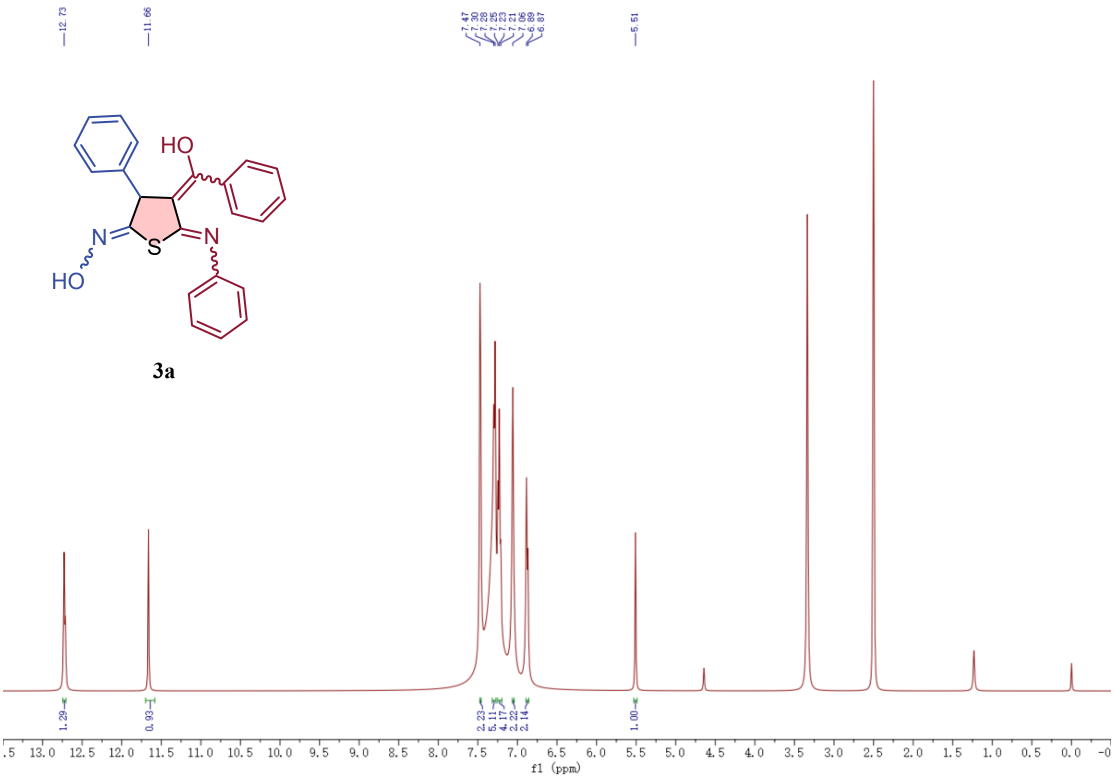

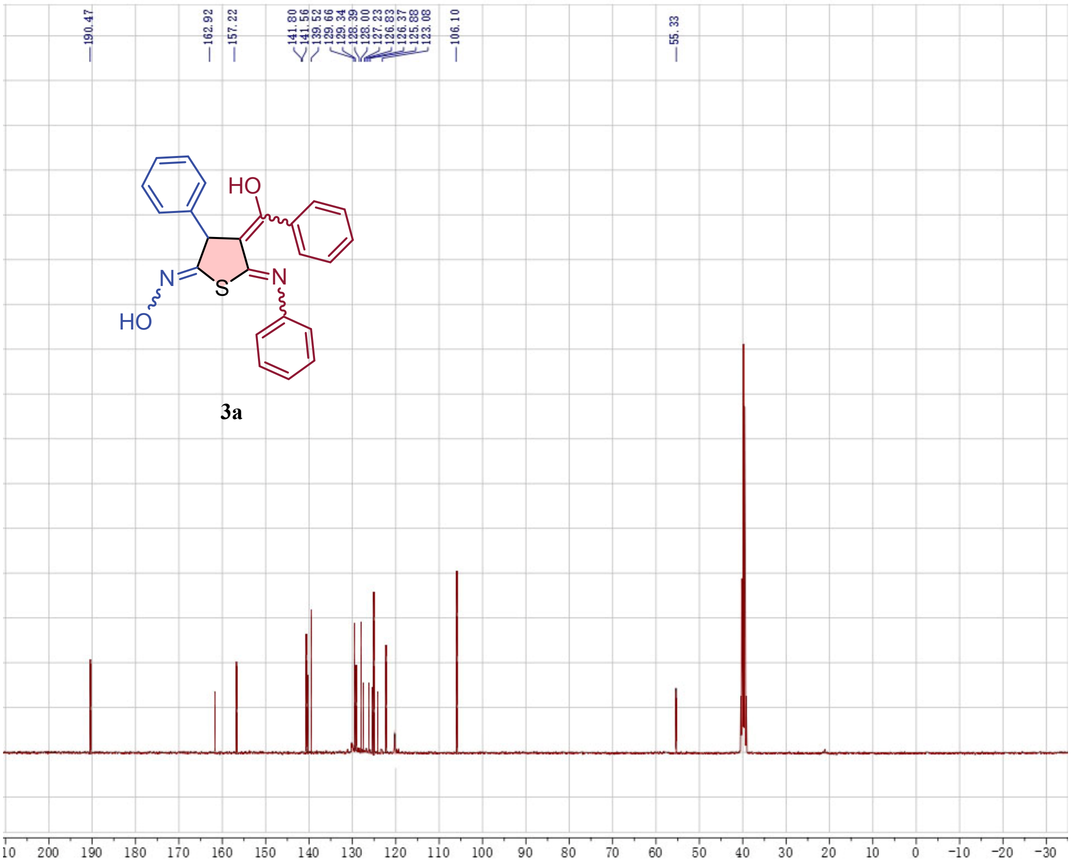

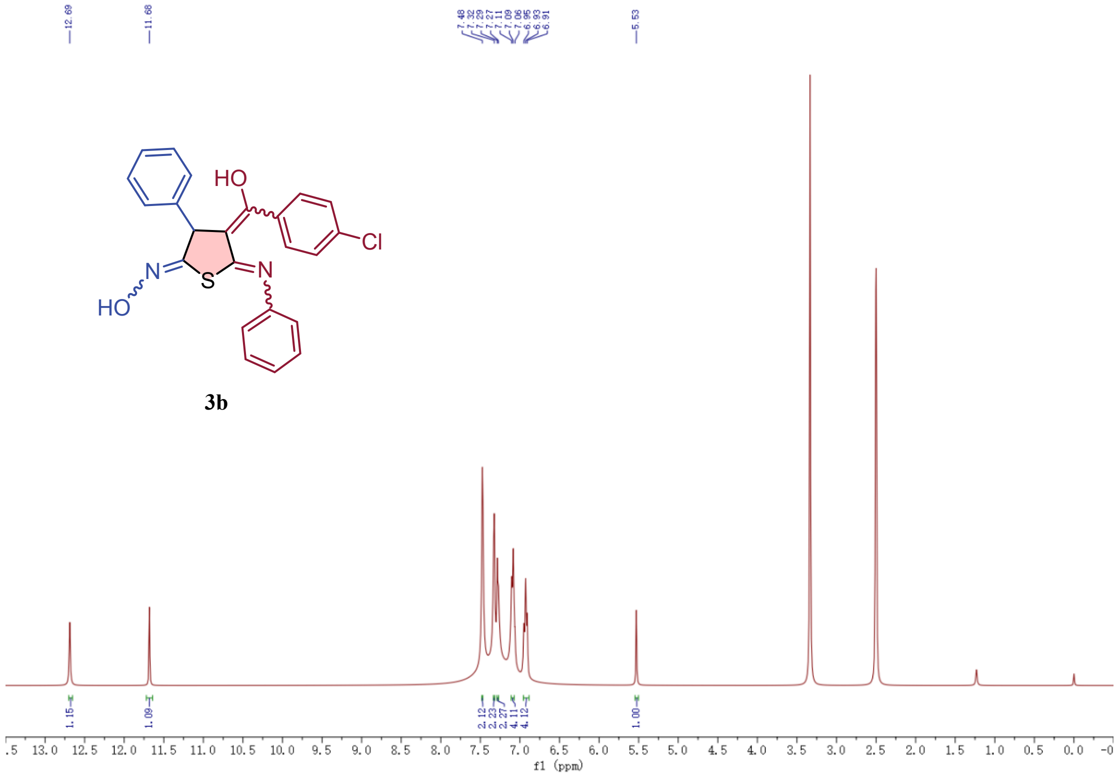
12.69
11.68
7.48
7.32
7.29
7.27
7.11
7.09
7.06
6.95
6.93
6.91
5.53
HO
Cl
N
S
N
HO
3b
1.15
1.09
2.12
2.23
2.27
4.11
4.12
1.00
f1 (ppm)

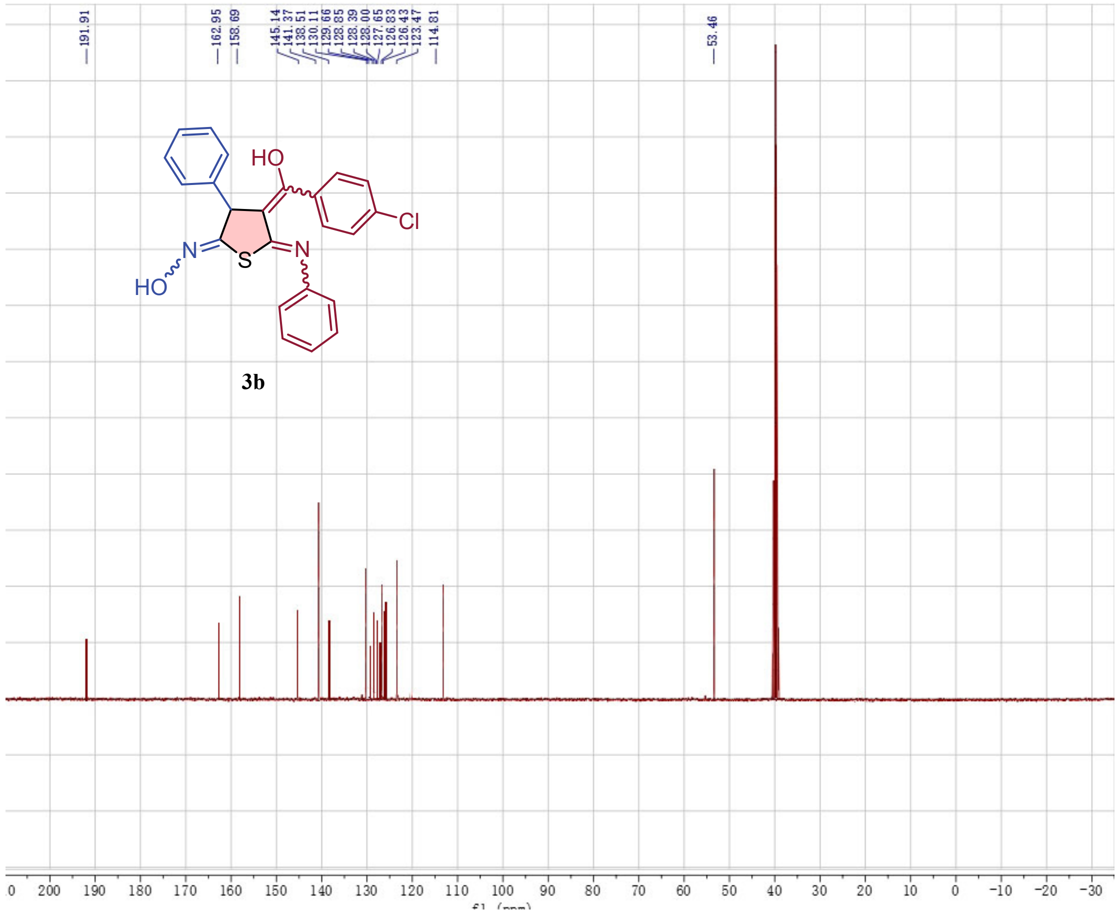
191.91
162.95
158.69
145.14
141.37
138.51
130.11
129.66
128.85
128.39
128.00
127.65
126.83
126.43
123.47
114.81
53.46
HO
Cl
N
S
N
HO
3b

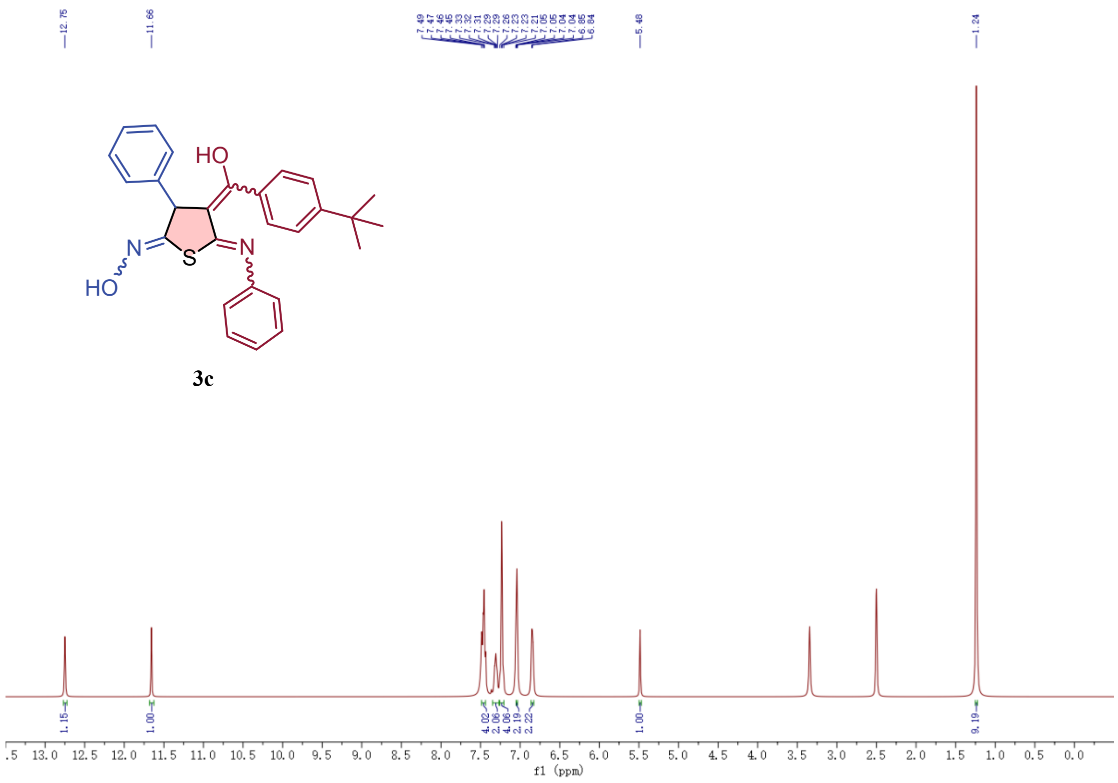

HO
N
S
HO
3c
12.75
11.66
5.48
1.24
1.15
1.00
9.19
f1 (ppm)

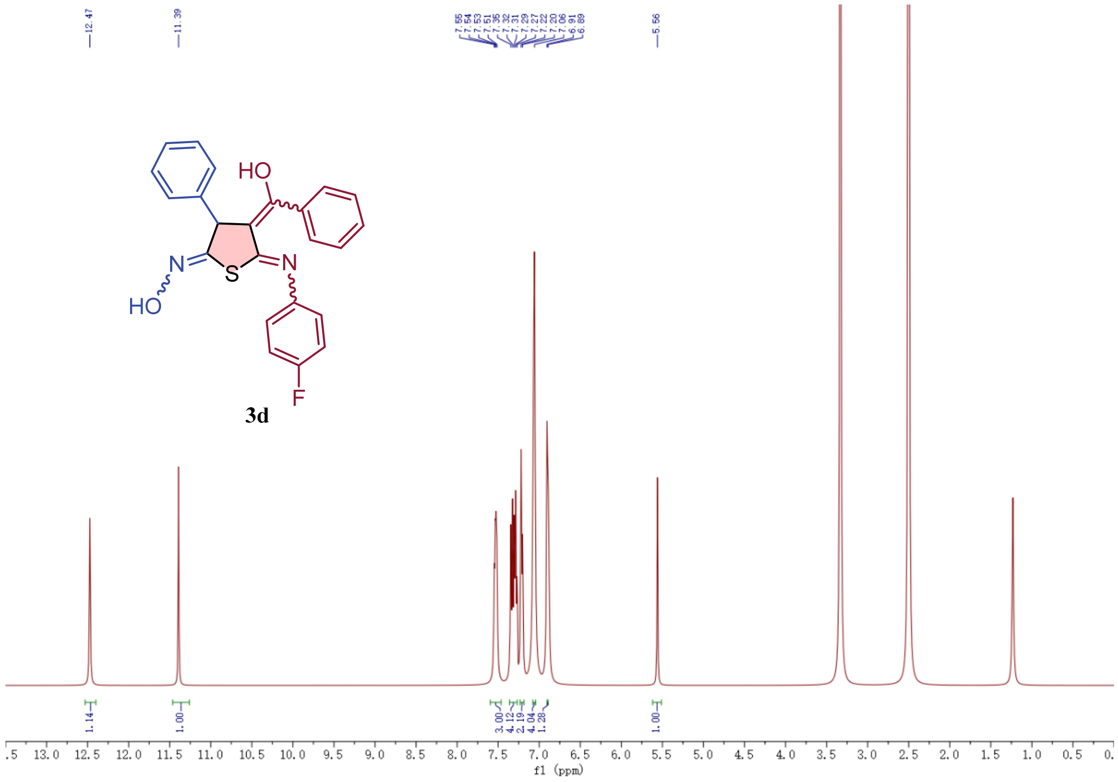

HO
N
S
HO
F
3d
12.47
11.39
5.56
1.14
1.00
f1 (ppm)

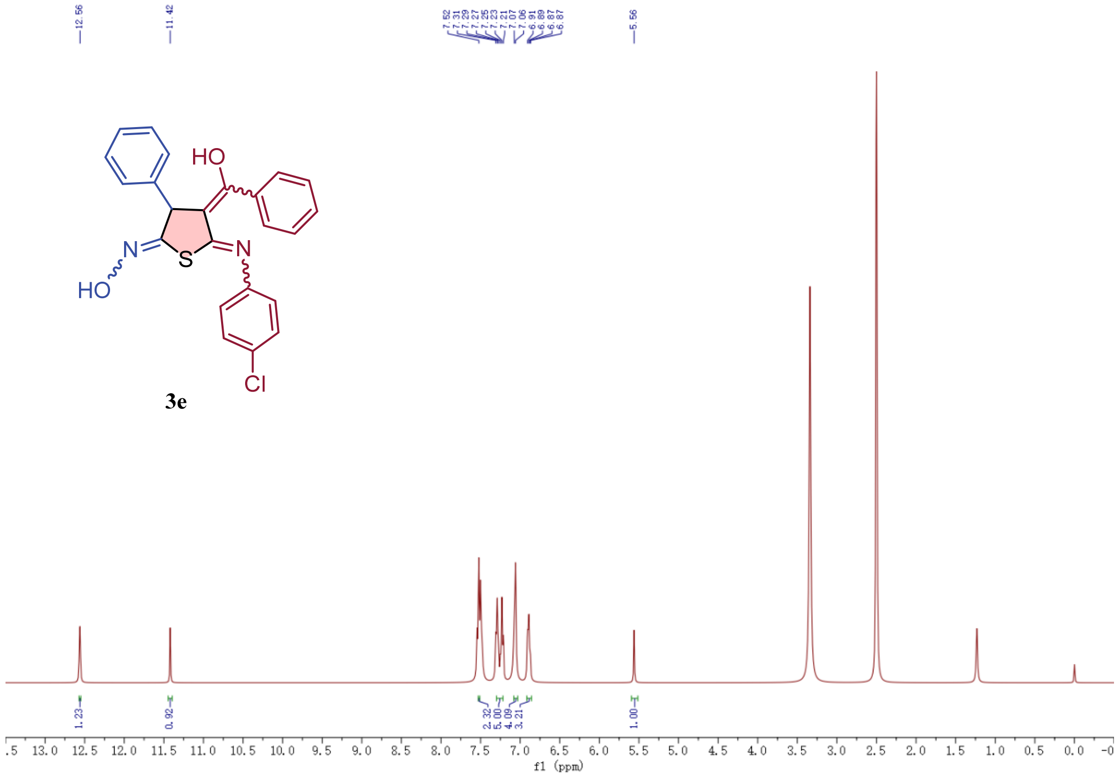
HO
N
S
N
HO
Cl
3e
f1 (ppm)

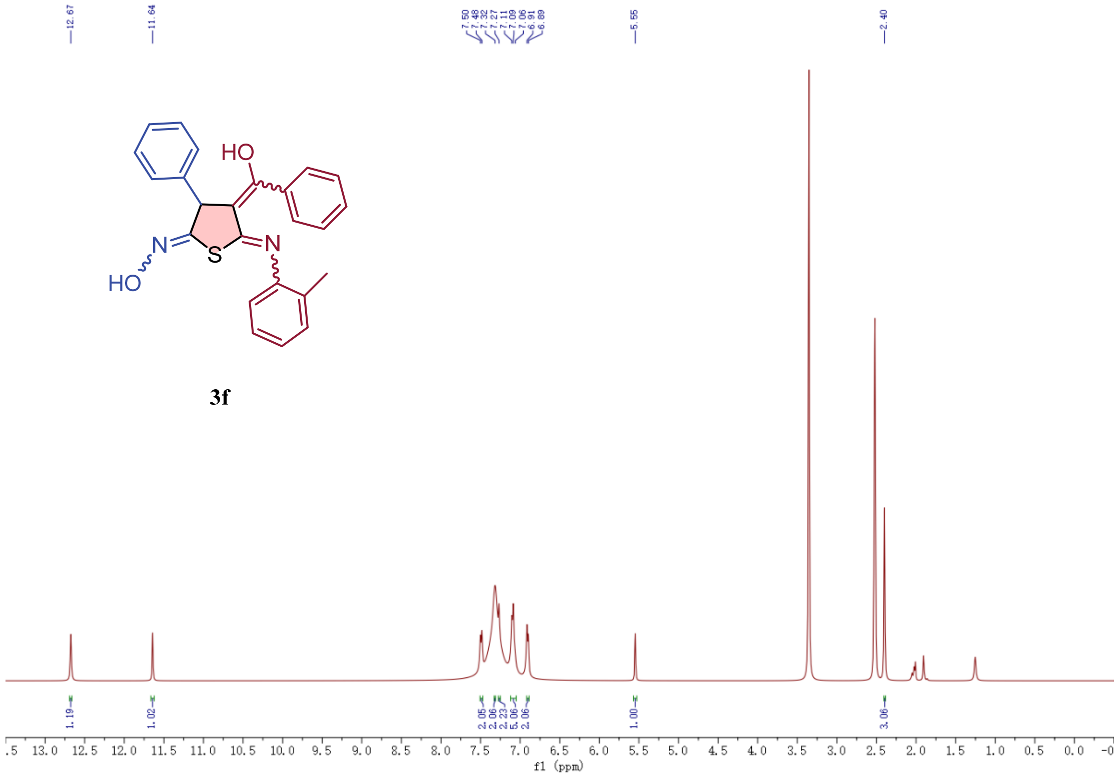
HO
N
S
N
HO
3f
f1 (ppm)

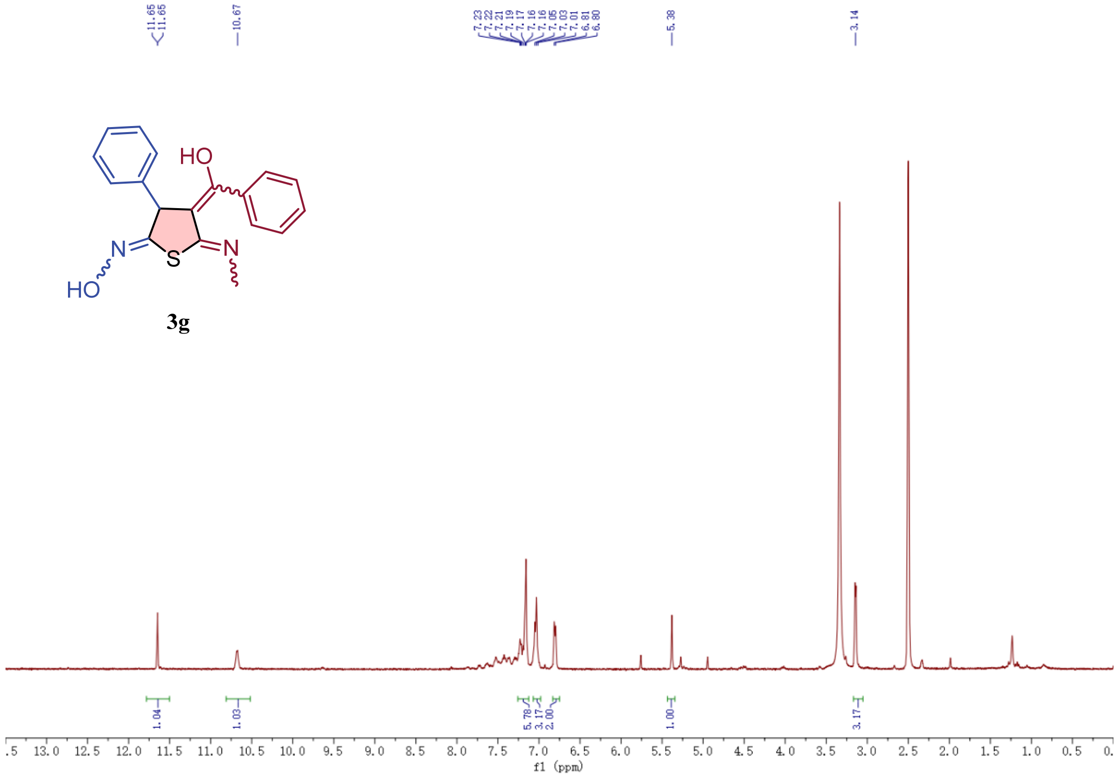
3g
f1 (ppm)

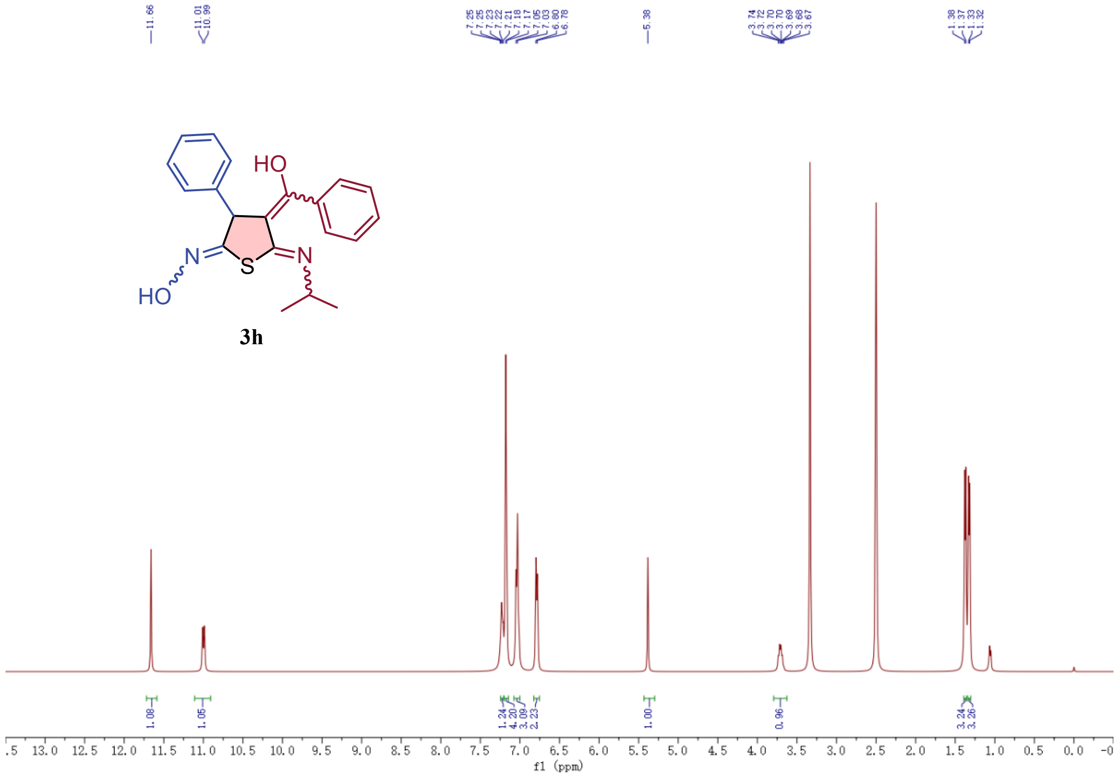
3h
f1 (ppm)

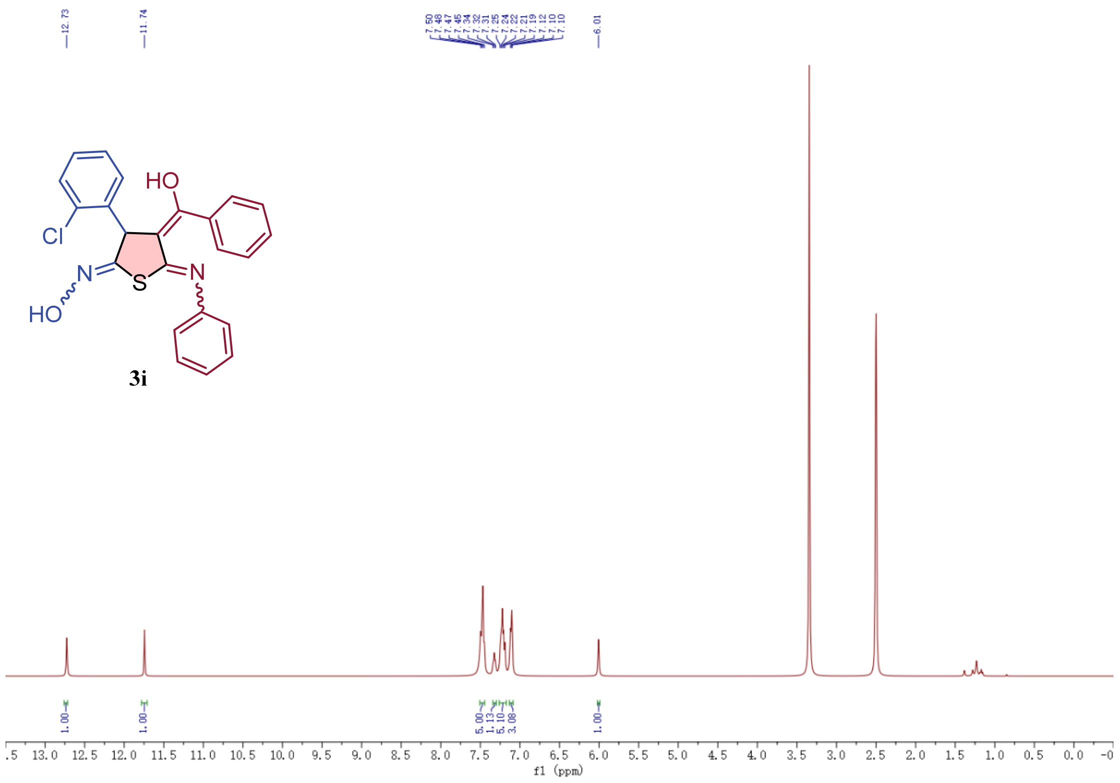
12.73
11.74
7.50
7.48
7.47
7.45
7.34
7.32
7.31
7.25
7.24
7.22
7.21
7.19
7.12
7.10
7.10
6.01
HO
Cl
N
S
N
HO
3i
1.00
1.00
5.00
1.13
5.10
3.08
1.00
f1 (ppm)

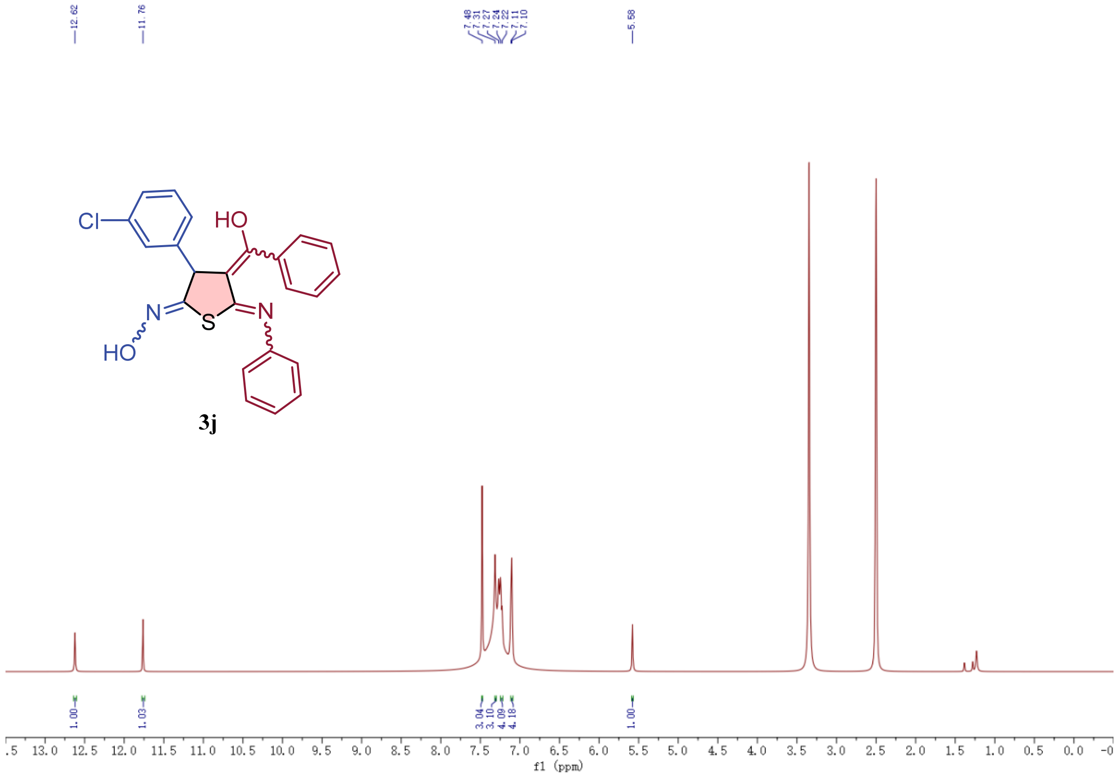
12.62
11.76
7.48
7.31
7.27
7.24
7.22
7.11
7.10
5.58
Cl
HO
N
S
N
HO
3j
1.00
1.03
3.04
3.10
4.09
4.18
1.00
f1 (ppm)

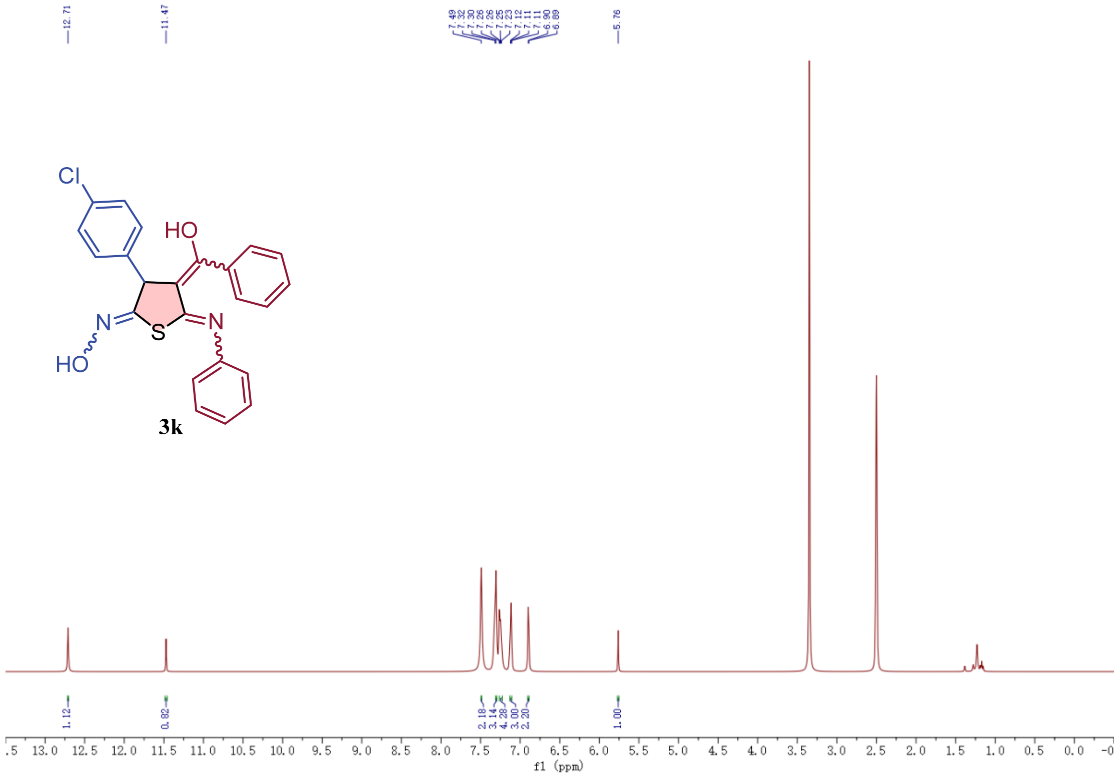
3k
f1 (ppm)

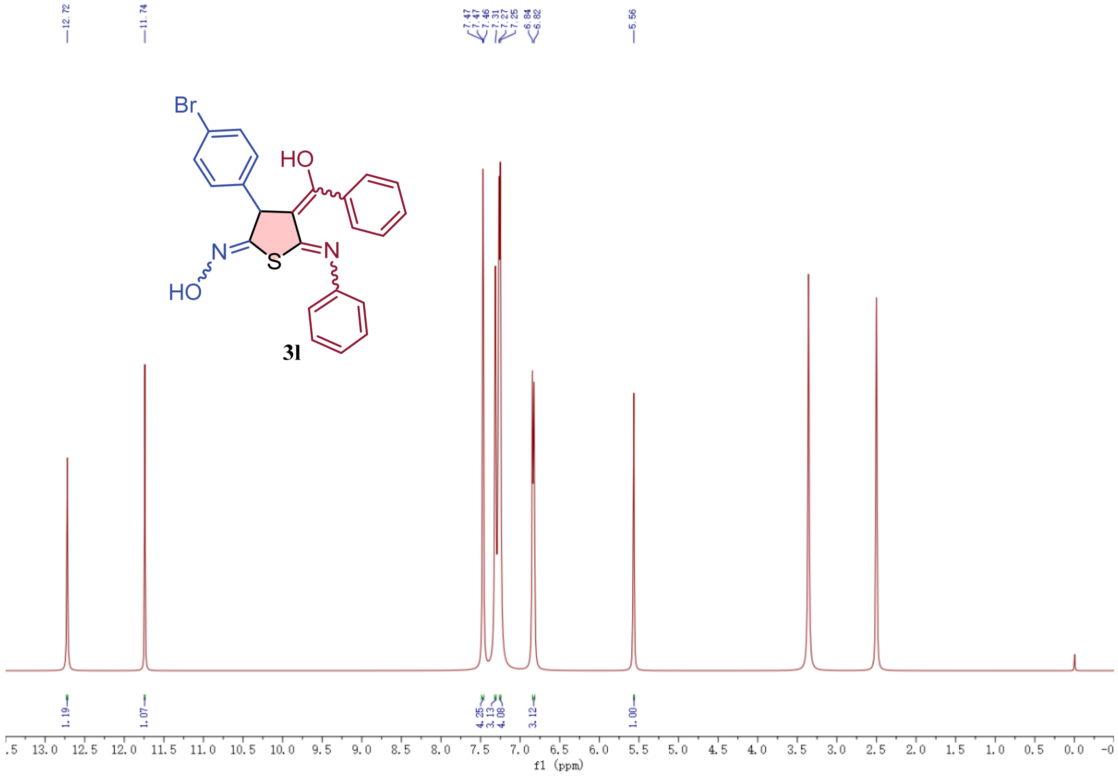
3l
f1 (ppm)

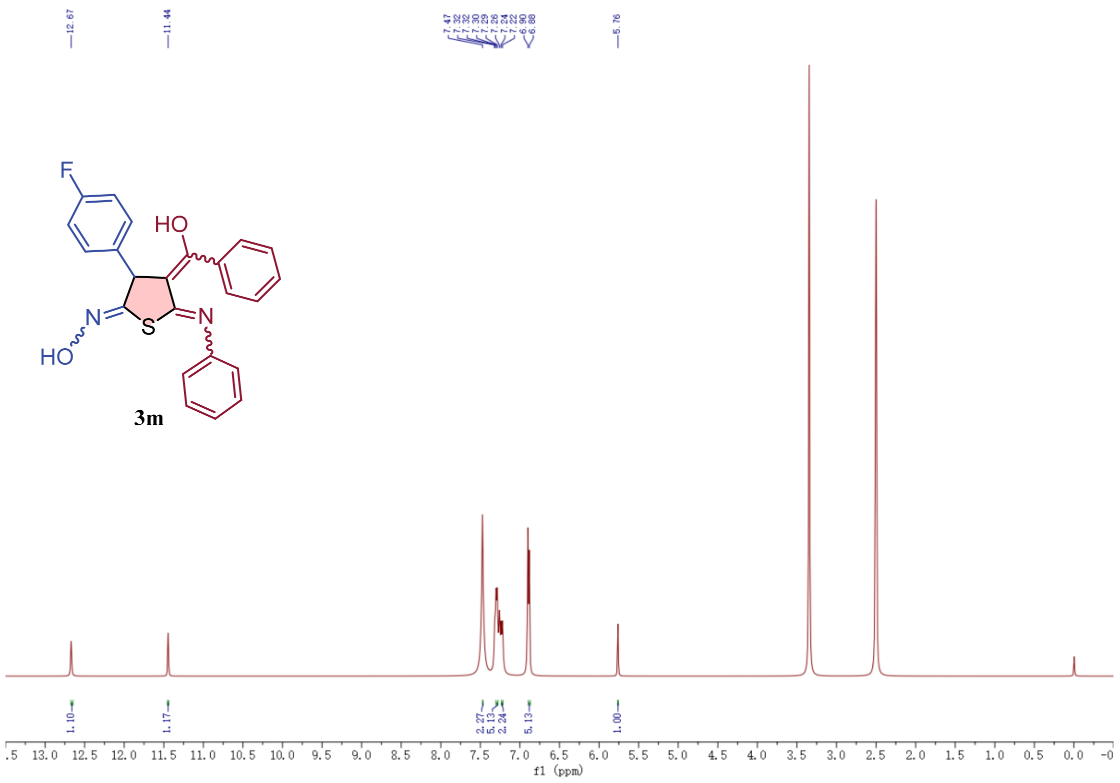
12.67
11.44
7.47 7.32 7.32 7.30 7.29 7.26 7.24 7.22 6.90 6.88
5.76
F
HO
N
S
N
HO
3m
1.10
1.17
2.27 5.13 2.24
5.13
1.00
f1 (ppm)

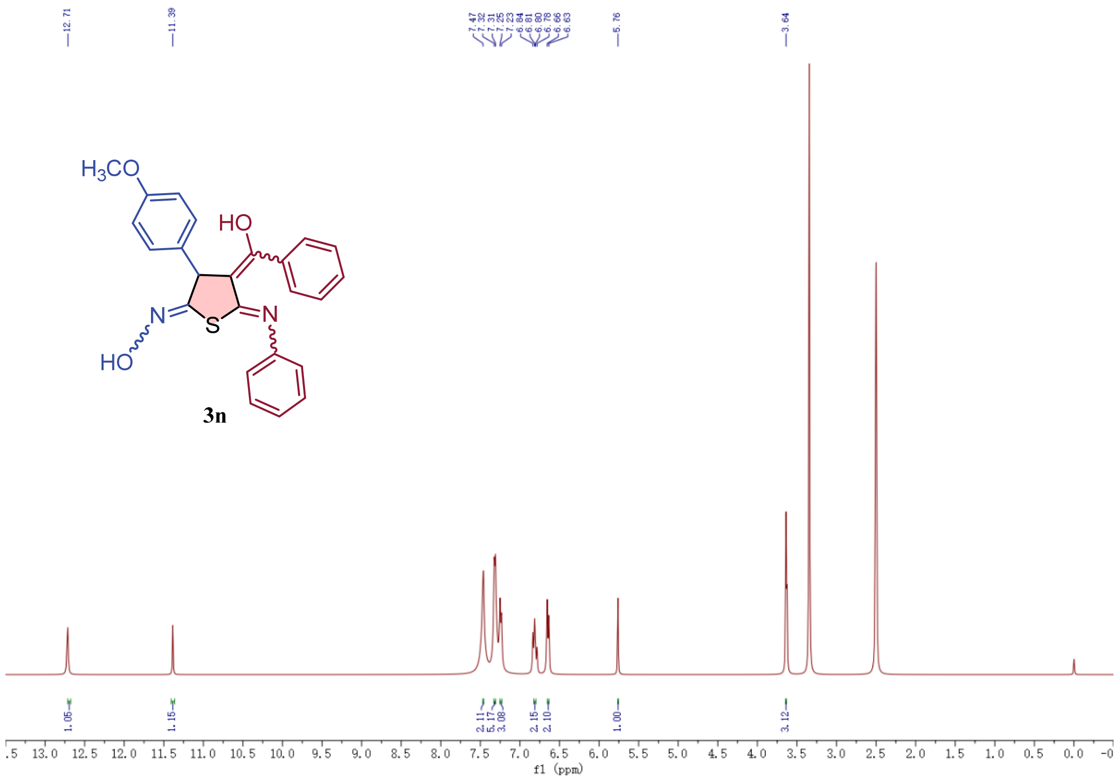
12.71
11.39
7.47 7.32 7.31 7.25 7.23 6.84 6.81 6.80 6.78 6.66 6.63
5.76
3.64
H_3CO
HO
N
S
N
HO
3n
1.05
1.15
2.11 5.17 3.08
2.15 2.10
1.00
3.12
f1 (ppm)

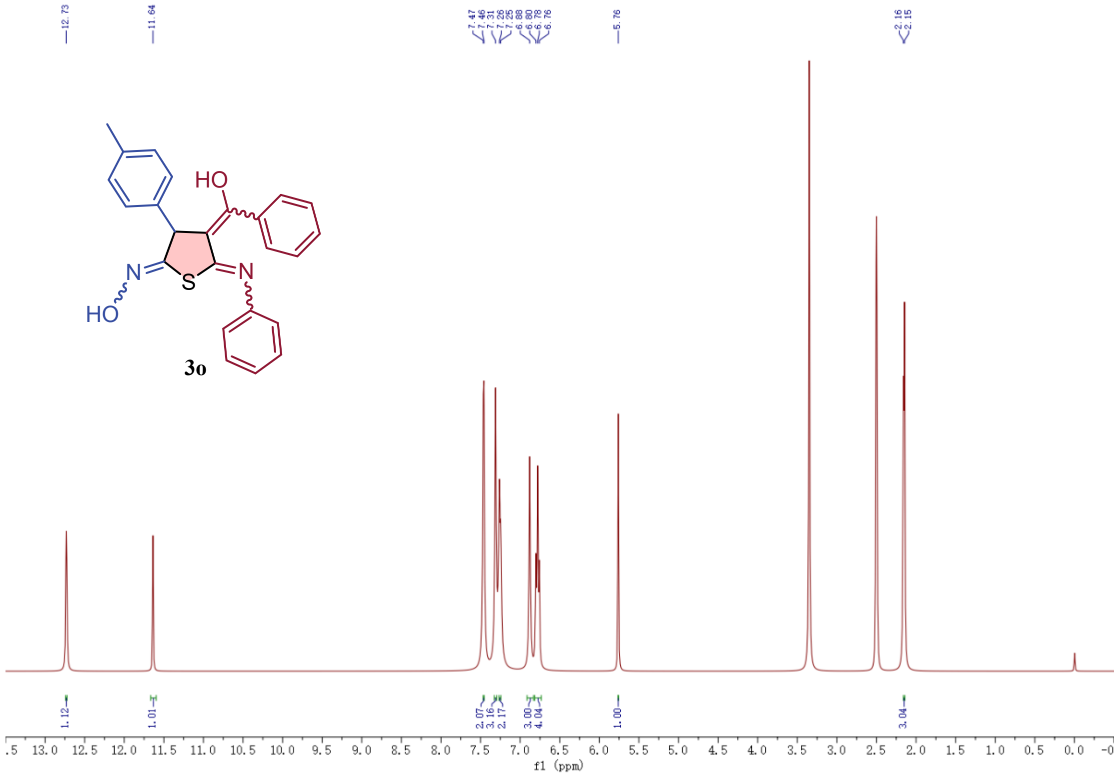
12.73
11.64
7.47
7.46
7.31
7.26
7.25
6.88
6.80
6.78
6.76
5.76
2.16
2.15
HO
N
S
HO
N
3o
1.12
1.01
2.07
3.16
2.17
3.00
4.04
1.00
3.04
f1 (ppm)

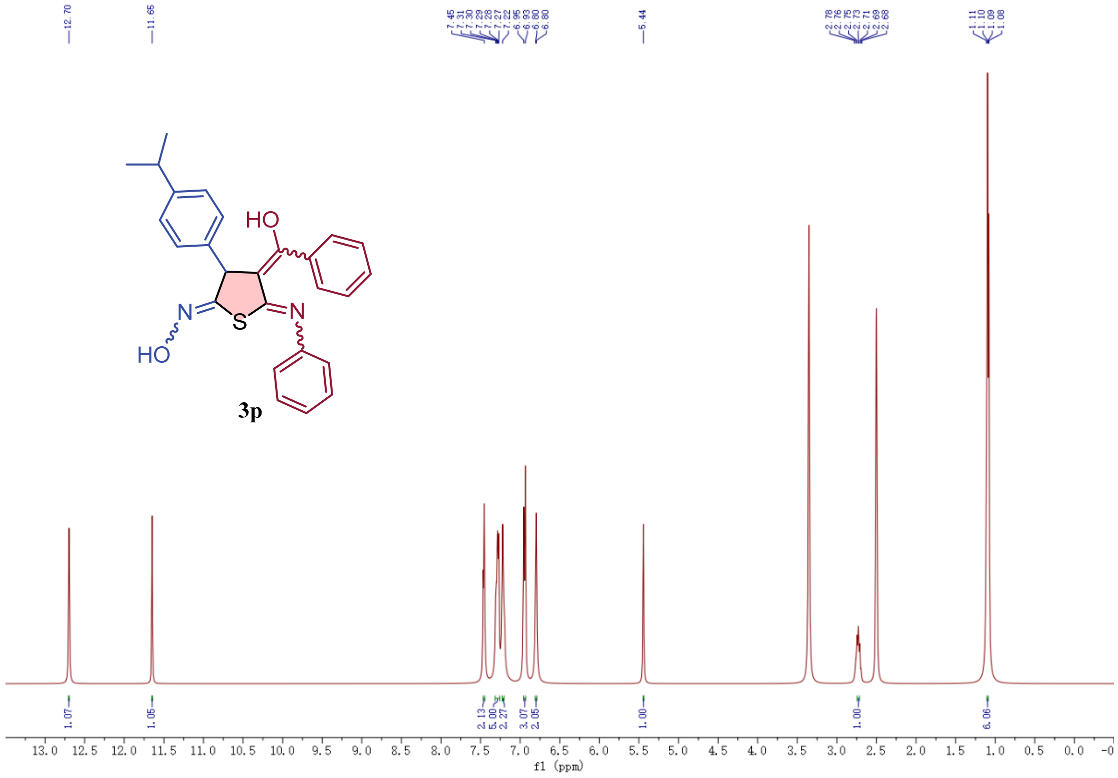
12.70
11.65
7.45
7.31
7.30
7.29
7.28
7.27
7.22
6.95
6.93
6.80
6.80
5.44
2.78
2.76
2.75
2.73
2.71
2.69
2.68
1.11
1.10
1.09
1.08
HO
N
S
HO
N
3p
1.07
1.05
2.13
5.00
2.27
3.07
2.05
1.00
1.00
6.06
f1 (ppm)

S3. Mass spectrum of 3a

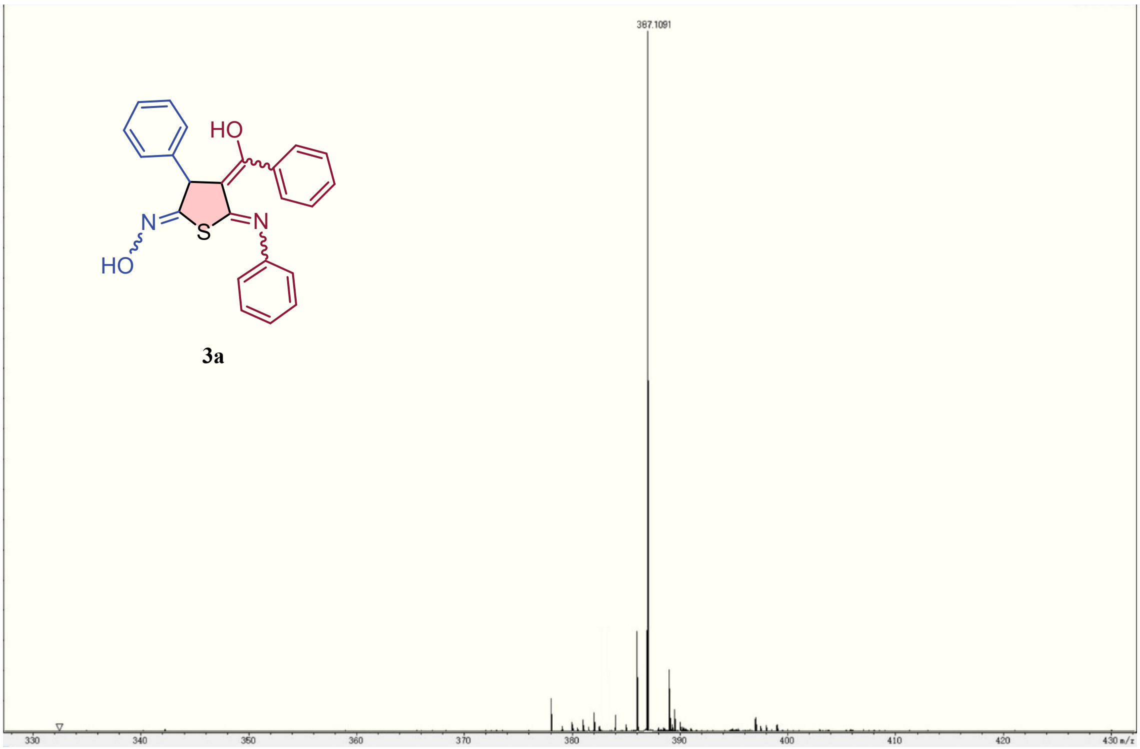

Supplement: Supplementary file 1 [file molecules-30-03202-s001.zip › molecules-3770150-supplementary.pdf]
